# Burnout dynamic among Ukrainian academic staff during the war

**DOI:** 10.1038/s41598-023-45229-6

**Published:** 2023-10-20

**Authors:** Natalia Tsybuliak, Yana Suchikova, Liudmyla Shevchenko, Anastasia Popova, Serhii Kovachev, Olha Hurenko

**Affiliations:** 1 Berdyansk State Pedagogical University, Berdiansk, Ukraine; 2 Vinnytsia Mykhailo Kotsiubynskyi State Pedagogical University, Vinnytsia, Ukraine

**Keywords:** Psychology, Health care

## Abstract

This study examined burnout dynamics among Ukrainian academic staff during the full-scale war. A cross-sectional study was conducted twice: the first wave in July 2022 and the second in January 2023. To assess the burnout syndrome as the final outcome, the self-reported Maslach Burnout Inventory-Human Services adapted for socioeconomic professions by Vodopyanova was used and correlated with different characteristics. The results showed a significant positive trend in emotional exhaustion among Ukrainian academic staff, with females being more sensitive to war-related factors. The results demonstrate that prolonged stressful situations associated with the ongoing war and constant changes in professional conditions lead to significant depersonalization dynamics among academic staff of both sexes. For male academic staff, factors such as age and academic position become less significant for depersonalization. However, university relocation and migration processes were significant factors affecting female academic staff’s' perception of effectiveness and accomplishment in their professional activities. The alarming dynamic of burnout levels detected among Ukrainian academic staff urges the national and institutional levels to take prompt actions to enhance the academic staff’s mental health in the workplace for preserving not only the quality of higher education, but also human capital in war times for postwar recovery in Ukraine.

## Introduction

Burnout is recognized as a common professional hazard in various people-centric professions^1,2^. Professionals in the education sector are particularly vulnerable to an increased risk of burnout, as teaching inherently involves demanding working conditions^3^. According to Karasek's theory, teaching is classified as a stressful profession^4,5^. Significant proportion of academic staff rate their work as the primary source of stress^6,7^.

Over the past several decades, research focusing on the mental health of academic staff has consistently reported that academic staff feel work overload and perpetually grapple to meet the exigencies of the academic environment^4,8–10^. The situation is further compounded by universities' inability to foster work environments with minimized stress levels^11^. Political shifts and transformations exert additional pressure on the staff within higher education institutions^12^.

The educational landscape is characterized by intense professional competition, lofty societal expectations, demands from administrative bodies and students^13^, constant institutional and organizational modifications^14^, substantial workloads^15^, high publication pressure^16,17^, teaching–research conflict^18,19^, the necessity of managing relationships with a multitude of students, colleagues and administrative personnel^20^, and securing grant funding and undertaking research^21^, among others. Collectively, these factors serve as catalysts for chronic stress among academic staff.

On the other hand, research shows that academic staff do not receive sufficient support from the institutions where they work, against the backdrop of increased academic pressure and demands on them. Constant work contributes to the emergence of family conflicts that provoke greater frustration in basic needs^4,22^. This forms an attitude that individual progress will never be enough^23^. All of these factors provoke chronic stress and exhaustion due to the prolonged impact of work-related problems and the absence of sufficient motivation, the high level of which impedes exhaustion^24^. This leads to the psychosocial syndrome of professional burnout, which is manifested through overwhelming exhaustion, feelings of cynicism and detachment from the job, and a sense of ineffectiveness and lack of accomplishment^1^.

Interestingly, even pursuing "work-life balance" strategies, in which higher education professionals attempt to balance conflicting job demands, results in a sustained mental workload^25^. This, in turn, has a reciprocal effect: a positive impact on burnout and a negative impact on academic efficiency^26^.

Crisis events have a significant impact on the mental health of academic staff, serving as additional stressors that influence the prevalence of burnout. For instance, during the recent global crisis incited by the COVID-19 pandemic, the already concerning prevalence of burnout syndrome was further exacerbated^27^. Academic staff faced emergent challenges and stress factors, including limitations on accessing data and participants within teaching and learning, disruption of scientific support networks, restricted access to institutional resources, and suboptimal work-life balance^28,29^. In addition, a new form of fatigue emerged, specifically associated with the widespread use of technology and video conferencing platforms, termed "online fatigue". This phenomenon further intensified distress related to the pandemic and the manifestation of mental health problems^30^.

The impact of crises and extreme situations on the process of professional burnout, particularly war, is an important factor to consider in the context of studying. Notably, the influence of war serves as an additional factor in causing burnout^31^. Currently, we are witnessing the most extensive military conflict in Central Europe since 1945, instigated by the Russian Federation against Ukraine^32^. There is a lack of research examining the impact of the Russo-Ukrainian conflict on the mental health of academic staff. However, available studies indicate that over half of Ukrainian educators struggle with depression and emotional exhaustion^33^.

Ukrainian academic staff are courageously trying to hold their educational and scientific front^34,35^. However, despite their courage and desire to work, Ukrainian educators staff have been facing constantly changing conditions since February 24, 2024, which directly affect their professional activity. Today, the professional activity of a Ukrainian academic staff depends on the map of active hostilities, missile attacks, air raid alerts, the availability of electricity, and the stability of the Internet^36^.

Further analysis of academic publications reveals that, in addition to the insurmountable external factors, universities had to overhaul their management, teaching, and learning processes due to the devastating full-scale invasion. These alterations were dependent on the university locations, staff, and students and had an impact on academic staff, resulting in amplified responsibilities and a need for rapid adjustment to course content and teaching methodologies tailored to the individual abilities of each student^36–38^.

The ongoing military operations in Ukraine have necessitated an increase in universities' third mission, requiring academic staff to engage in volunteering activities beyond their professional workload^39–41^. Additionally, a significant number of academic staff members have been forced to flee their homes due to the occupation of their hometowns or escalating military activities. As a result, they have sought refuge in safer regions of Ukraine or abroad, causing additional personal tribulations and stress^34,42^. Some members of academic staff have been, or continue to be, subjected to relentless attacks by occupiers while residing in occupied territories^43,44^. These challenges represent only a fraction of the formidable obstacles that Ukrainian academic staff members are facing more than a year.

Despite these extraordinary circumstances, in the midst of full-scale war, universities and academic staff persist in fulfilling their main mission of delivering high-quality, modern education, steadfastly holding their line. However, it is crucial to investigate the condition and dynamics of burnout experienced in a state of unremitting chronic stress, caused by both the conventional challenges faced by academic staff and additional factors induced by warfare.

Research on this subject could generate significant international interest in gaining a deeper understanding of the current situation in Ukraine's higher education system. Additionally, this research would address a crucial and unique gap in studies regarding the mental health of academic staff, with a particular focus on burnout in crisis scenarios under war conditions. Our objective is to investigate the dynamics of burnout detected among Ukrainian academic staff and link it with factors associated with the ongoing full-scale war. The results of this research could potentially inform interventions designed to support academic staff's mental health in similarly challenging contexts.

## Results

A total of 836 academic staff members participated in the first wave of evaluation, whereas 228 participated in the second wave (Table 1). Women constituted the majority of respondents, accounting for 80% in the first wave and 86% in the second wave. In the first wave of the survey, 37% of the participants were migrants, with 13% of them having moved abroad. In the second wave, 32% of the participants were migrants, out of which 11% were located overseas. Additionally, 21% and 23% of respondents in the first and second waves, respectively, were from universities that had relocated from occupied territories to areas under Ukrainian control.

**Table 1:** Sociodemographic characteristics of evaluated Ukrainian academic staff.

Variable	Subcategory	1st wave		2nd wave	
		%	N	%	N
Age	Under 35	15	127	15	35
	35–50 years	45	379	37	84
	51–60 years	33	275	42	95
	61 older	7	55	6	14
Gender	Male	20	171	14	31
	Female	80	665	86	197
Scientific degree	Doctor of science	18	152	18	42
	PhD degree	64	536	67	152
	Magister degree	18	148	15	34
Academic position	Professor	18	147	18	41
	Associate professor	55	464	61	139
	Senior lecturer	18	148	15	34
	Assistant	9	77	6	14
Change of permanent residence	Internal military migrants	24	199	22	50
	External military migrants	13	111	11	24
	Remained in the places of permanent residence	63	526	68	154
Currently University location	Remained at a permanent location	79	659	77	176
	Temporarily relocated to the Ukraine-controlled territory	21	177	23	52

While studying the dynamics of burnout among Ukrainian academic staff during the full-scale war, significant changes were identified that occurred between July 2022 and January 2023 (Table 2).

**Table 2:** Comparison of the means of the burnout predictors among Ukrainian academic staff during the war.

Burnout predictors	Level	Emotional exhaustion				Depersonalization				Personal accomplishment			
		M1^a^	M2^b^	W1^c^	W2^d^	M1^a^	M2^b^	W1^c^	W2^d^	M1^a^	M2^b^	W1^c^	W2^d^
Age	Low	99.57	16	350.61	105.78	92.18	19.25	336.96	93.48	91.88	16.1	336.96	103.39
	Moderate	75.84	12.83	333.6	99.37	88.82	14.61	338.87	103.87	81.84	13.21	338.87	93.54
	High	80.93	17.27	327.76	97.44	61.85	15.28	308.51	98.21	80.78	19.61	308.51	104.45
Scientific degree	Low	87.48	12.6	327.29	99	86.14	10.75	319.52	106.78	85.1	11.6	319.52	104.46
	Moderate	83.06	17.67	333.1	112.02	85.89	18	338.73	100.02	85.62	18.5	338.73	99.91
	High	87.16	17.6	334.58	93.51	85.93	17.56	341.68	87.83	90.8	17.56	341.68	82.4
Academic Position	Low	79.93	11.7	342.76	93.74	84.12	10	306.76	104.5	80.69	12.95	306.76	105.97
	Moderate	87.79	20.58	331.18	113.5	88.43	17.54	345	102.07	87.67	18.25	345	98.93
	High	90.82	17.03	330.99	93.98	84.69	18.94	347.43	87.45	98.88	16.39	347.43	81.53
Currently University location	Low	84.78	16.4	296.66	89.98	83.09	13.69	322.91	92.26	83.09	14.85	322.91	100.76
	Moderate	90.09	11.75	322.4	98.32	88.55	16.18	336.81	102.04	87.65	16.92	336.81	91.19
	High	83.5	17.43	347.67	101.15	87.17	17.78	340.89	102.56	90.18	16.06	340.89	118.22
Change of permanent residence	Low	83.12	15.65	333.59	101.98	82.71	18.75	328.36	107.14	86.45	17.2	328.36	93.85
	Moderate	84.41	18.75	324.85	102.55	89.66	15.43	337.49	98.32	84.08	14.88	337.49	108.4
	High	90.56	15.13	336.2	96.89	85.28	14.44	328.67	90.04	91.85	16.17	328.67	83.55

^a^M1—Mean rank among men, the first wave of the study (5 months after the full-scale war).

^b^M2—Mean rank among men, the second wave of the study (11 months after the full-scale war).

^c^W1—Mean rank among women, the first wave of the study (5 months after the full-scale war).

^d^W2—Mean rank among women, the second wave of the study (11 months after the full-scale war).

**Figure 1:**
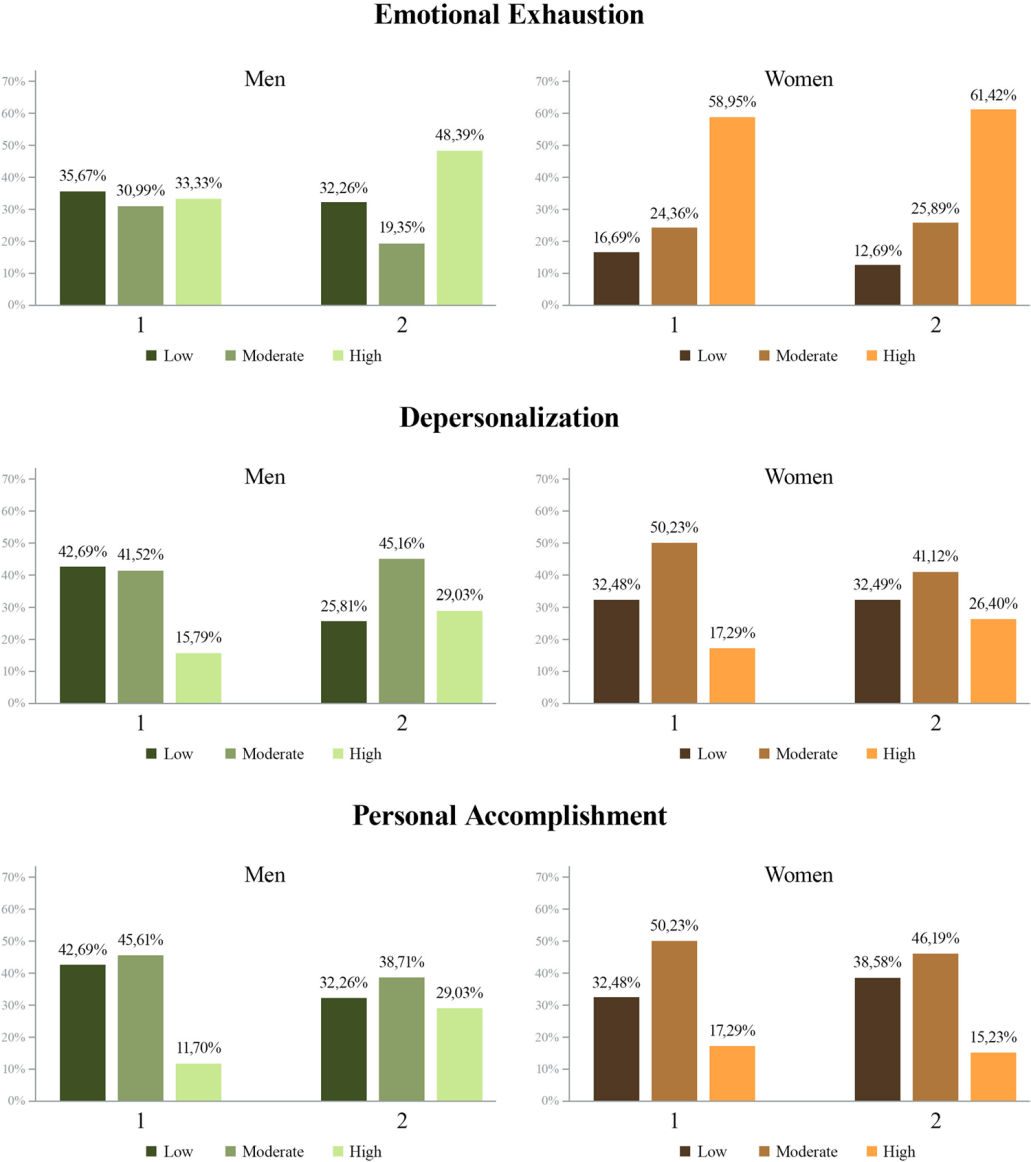
The dynamic burnout prevalence among Ukrainian academic staff.

In terms of emotional exhaustion, it was discovered that the proportion of individuals experiencing high levels of emotional exhaustion increased for both men and women. Specifically, for men, the percentage of academic staff experiencing high levels of emotional exhaustion rose from 33.33% in July to 48.39% in December. Similarly, for women, this percentage increased slightly from 58.95% to 61.42%. There was also a significant increase in the percentage of individuals experiencing high levels of depersonalization for both genders. Among men, the percentage of academic staff exhibiting high levels of depersonalization increased from 15.79% in July to 29.03% in December. Women also showed an increase from 17.29% to 26.40%. With regard to personal accomplishments, there was a decrease in the percentage of individuals with high levels for both sexes. For men, the percentage of those with low levels of personal achievement decreased from 42.69% to 32.26%. However, among women, there was an increase in indicators of low levels of this predictor: from 32.48% to 38.58% (Fig. 1).

We conducted a separate analysis to examine the relationship between burnout predictors (emotional exhaustion, depersonalization, personal accomplishment) and age, academic degree, job position, current university location, and change of permanent residence since the onset of the full-scale war in Ukraine for both men and women during the 1st and 2nd waves (as shown in Table 3).

**Table 3:** Association between burnout predictors with socio-demographic characteristics.

Burnout predictor	Gender	Wave	Age		Scientific degree		Academic position		Change of permanent residence		Currently University location	
			Chi-Square (χ^2^)	Asymp. Sig.	Chi-Square (χ^2^)	Asymp. Sig.	Chi-Square (χ^2^)	Asymp. Sig.	Chi-Square (χ^2^)	Asymp. Sig.	Chi-Square (χ^2^)	Asymp. Sig.
EE	Men	1	8.056	0.018	0.343	0.843	1.77	0.413	1.14	0.565	1.406	0.495
		2	1.114	0.573	2.612	0.271	4.779	0.092	3.244	0.197	1.067	0.587
	Women	1	1.446	0.485	0.177	0.915	0.43	0.807	13.33	0.001	0.519	0.771
		2	0.518	0.772	5.639	0.06	5.944	0.051	1.522	0.467	0.633	0.729
DP	Men	1	8.473	0.014	0.001	0.999	0.342	0.843	0.953	0.621	1.354	0.508
		2	1.539	0.463	4.565	0.102	5.833	0.054	1.651	0.438	1.601	0.449
	Women	1	2.689	0.261	2.992	0.224	2.162	0.339	12.856	0.002	2.088	0.352
		2	1.394	0.498	4.796	0.091	3.957	0.138	2.512	0.285	3.813	0.149
PA	Men	1	1.959	0.375	0.273	0.872	2.645	0.266	1.008	0.604	0.759	0.684
		2	2.791	0.248	4.445	0.108	2.268	0.322	0.538	0.764	0.55	0.76
	Women	1	2.682	0.262	2.271	0.321	7.473	0.024	1.818	0.403	0.475	0.789
		2	1.81	0.405	4.868	0.088	5.278	0.071	9.811	0.007	7.762	0.021

*EE* emotional exhaustion, *DP* depersonalization, *PA* personal accomplishment.

The analysis of the results from the first wave of the independent variable "Age" indicates a statistically significant difference in emotional exhaustion (EE) for men, χ21mEE = 8.056, p1mEE = 0.018. The average rank score for low, moderate, and high levels of EE were 99.57, 75.84, and 80.93, respectively. Additionally, there was a statistically significant difference in depersonalization (DP) for men, χ^2^1mDP = 8.473, p1mDP = 0.014. The average rank score for low, moderate, and high levels of DP were 92.18, 88.82, and 61.85, respectively. However, during the second wave for men and both waves for women, no statistically significant differences were found.

The results of the first wave of the independent variable "Academic Position" indicate that there is a statistically significant difference in Personal Achievement (PA) for women (χ^2^1wPA = 7.473, p1wPA = 0.024), with an average rank score of 306.76 for low, 345 for moderate, and 347.43 for high levels of PA. The second wave of emotional exhaustion (EE) for women also showed a statistically significant difference (χ^2^2wEE = 5.944, p2wEE = 0.051), with an average rank score of 342.76 for low, 331.18 for moderate, and 330.99 for high levels of EE. Additionally, Depersonalization (DP) for men had a statistically significant difference (χ^2^2mDP = 5.833, p2mDP = 0.054), with an average rank score of 10 for low, 17.54 for moderate, and 18.94 for high levels of DP. No statistically significant differences were found for the remaining indicators.

The analysis of the results from the first wave of the independent variable "Current University Location" indicates a statistically significant difference in emotional exhaustion (EE) for women, χ^2^1wEE = 13.33, p1wEE = 0.001. The average rank score for low, moderate, and high levels of EE were 296.66, 322.4, and 347.67, respectively. Additionally, depersonalization (DP) in women showed a significant difference, χ^2^1wDP = 12.856, p1wDP = 0.002, with an average rank score of 322.91 for low, 336.81 for moderate, and 340.89 for high levels of DP. The second wave of personal achievement (PA) for women also showed a significant difference, χ^2^2wPA = 9.811, p2wPA = 0.007, with an average rank score of 100.76 for low, 91.19 for moderate, and 118.22 for high levels of PA. No statistically significant differences were found for the remaining indicators in women or both waves for men.

The results of the second wave of the independent variable "Change of Permanent Residence" indicate that there is a statistically significant difference in personal achievement (PA) for women (χ^2^2wPA = 7.762, p2wPA = 0.021). The average rank score for low, moderate, and high levels of PA were 93.85, 108.4, and 83.55 respectively. However, no statistically significant differences were observed for the remaining indicators.

## Discussion

The present study reveals the impact of the ongoing full-scale war on the mental health of Ukrainian academic staff by examining the factors related to burnout. Sociodemographic factors were analyzed.

The results of both surveys conducted among Ukrainian academic staff of both genders demonstrate a significant positive trend in emotional exhaustion, indicating the emotional burden that they must cope with during times of war. It is interesting to note that the level of emotional exhaustion is significantly higher among female academic staff in both the first and second surveys. This finding is generally confirmed by many studies that discuss the difference in this burnout predictor between male and female representatives^]^. However, our study reveals an interesting observation that among male academic staff, a significant positive trend in emotional exhaustion is observed over a year of full-scale war. In contrast, among female academic staff, this growth indicator is not as significant. This difference may be explained by the fact that women tend to manage stress by communicating with others in their lives, while men tend to internalize stress^48^. Our study shows that the longer the stress at the workplace persists during a state of war, the less the age of male academic staff affects emotional exhaustion. Consequently, teaching in the conditions of a full-scale war, which changes teaching and learning approaches and practices, is a challenge for male academic staff who struggle to adapt to new realities.

The results of the study demonstrate that prolonged stress related to the ongoing war and increased workload leads to significant depersonalization dynamics among academic staff of both genders. Previous research has indicated that depersonalization correlates with factors such as job satisfaction, internal teaching orientation, interpersonal activity in the classroom, and teacher-student relations^49–51^. However, a crisis situation such as war can alter the impact of these factors. Specifically, our study shows that for male academic staff, the impact of factors on depersonalization changes. For example, the first survey reveals a correlation between depersonalization in male academic staff and age, while the second survey shows that this factor becomes insignificant for burnout, and academic position becomes significant. The situation is different among female academic staff. The first survey indicates the influence of university relocation during a full-scale war to a new location in Ukraine-controlled territory. However, the second survey does not show such a connection, indicating that regardless of age, academic position, scientific degree, university relocation, and migration processes, female academic staff in conditions of full-scale war feel alienated from work, cynical, and detached from their professional activity.

The research results indicate that female academic staff experience a reduction in accomplishment during the ongoing war in Ukraine. In the first survey, the academic position was found to be a significant factor influencing the perception of personal accomplishment among female educators. However, six months into full-scale war, this factor no longer has a significant impact. Instead, factors such as the university's relocation and migration processes among female academic staff become significant, affecting the adequacy of perception of effectiveness and accomplishment. Several studies on the impact of war on Ukrainian universities outline the problems encountered by Ukrainian academic staff after the relocation of the university to a new location and forced migration^52–55^.

Upon analyzing socio-demographic characteristics, we have found that age is a significant factor in influencing emotional exhaustion and depersonalization in male educators during the first few months of a full-scale war. However, as time passes, the significance of these factors changes and becomes non-essential for the burnout dynamic. Additionally, academic degree was found to be insignificant for burnout among Ukrainian academic staff during times of full-scale war, despite studies showing that academic staff who are just starting their professional journey are more exposed to stress factors in the workplace^56^.

It is interesting to note that the "Academic position" factor has an influence on the burnout dynamic among female and male academic staff. For female members of academic staff, this factor was significant depending on the duration of the full-scale war. In the first few months, there was a correlation between academic position and accomplishment. However, after almost a year of full-scale war, changes are observed. The academic position of females affects emotional exhaustion, whereas for males, their academic position affects the dynamics of depersonalization, depending on the duration of the war.

When evaluating war-related factors, we discovered that the prolonged situation affects predictors of burnout differently among the academic community of both genders. Specifically, the temporary relocation of the university to a new location significantly affects the emotional exhaustion and depersonalization of female academic staff in the first few months of a full-scale war. However, as this process continues, the nature of the influence of this factor changes, and it begins to affect the perception of personal accomplishment. Additionally, migration processes become an important factor. Furthermore, we found that female academic staff are more sensitive to the influence of war-related factors.

The alarming levels of burnout detected among Ukrainian academic staff represent only the tip of the iceberg of the mental health problems caused by the war. The negative impacts of the full-scale war may further provoke a change in the quality of the educational process in higher education institutions and the professional reorientation of academic staff. However, studies on the impact of a full-scale war in Ukraine on the mental health of various categories of the population are still unfolding. This also applies to tangible measures focused on burnout for various professions. In the field of higher education, it is important to address the mental health of academic staff right now in order to provide timely support and reduce the dynamics of burnout as much as possible. These benefits both academic staff and students, as well as the overall system of higher education. Therefore, it is important to address the factors identified by this study that potentially contribute to burnout among academic staff for the development of psychological support programs at both the institutional and state levels. However, these approaches need to be further explored.

## Conclusion

Scientific research analyzing the impact of full-scale war on the mental health of Ukrainian academic staff has shown that burnout among this group has increased due to the war. Female academic staff were found to be particularly vulnerable to the negative effects, experiencing greater emotional exhaustion and alienation from their work throughout the entire year of the war. Meanwhile, research suggests that the duration of the war more significantly impacts the emotional exhaustion of male academic staff. It was also found that the duration of the full-scale war affects the dynamics of depersonalization. In the case of male academic staff, factors such as age and academic position become less significant for depersonalization. However, for females, university relocation and migration processes proved to be significant factors affecting their perception of effectiveness and achievements in professional activity.

These findings emphasize the need for further research on the impact of war on academic staff burnout, as well as the development and implementation of appropriate support strategies and interventions. Burnout can have a negative impact on academic staff productivity and mental health, which ultimately affects the quality of education provided to students. Therefore, it is crucial to minimize this effect.

The alarming trend of burnout levels detected among Ukrainian academic staff highlights the need for prompt action at both the national and institutional levels to improve the mental health of academic staff. This is crucial for preserving human capital in the field of higher education during times of war and for postwar recovery. Further research can also help to identify the factors that increase or decrease the risk of burnout under these conditions, enabling the development of more targeted and effective interventions.

## Limitations

Our study has several significant limitations that must be considered. Firstly, the cross-sectional design of the study could have resulted in selection bias, which may impact the generalizability of our findings. Secondly, the online assessment utilized to gather data during the full-scale war may not be entirely reliable due to response bias. Therefore, it is crucial to exercise caution when interpreting our results as they do not serve as a clinical psychiatric diagnostic tool.

Furthermore, the data we collected relied heavily on self-reported information during a prolonged period of stress caused by the ongoing full-scale war in Ukraine. This reliance on self-reported information could have led to bias, thereby making it difficult to draw accurate conclusions.

Despite its limitations, the data collected is crucial in understanding the overall situation regarding burnout among academic staff during times of war. It can also help in developing a system of rapid response measures at different levels of the higher education system. We suggest conducting follow-up qualitative longitudinal studies to confirm our results, especially after Ukraine's victory in the war.

## Methods

### Study design and data collection

This study is a cross-sectional analytical research. Data was collected through an online survey. A preliminary instrument was tested on 15 academic staff to ensure question clarity and confirm the survey's completion within approximately 12 min. The research was conducted in two waves, the first in July 2022 (5 months after the full-scale war), and the second in January 2023 (11 months after the full-scale war). The questionnaire was distributed to Ukrainian university faculty by email using Google Forms for data collection.

Participation was both anonymous and voluntary throughout the study period. Participants were informed about the research and its goals before giving their consent. However, we were unable to assess the number of people who viewed the online invitation, and therefore, we could not determine the response rate of the study.

The inclusion criteria were academic staff who worked in Ukrainian universities during the full-scale war period. The exclusion criteria were incomplete questionnaires. Pilot test data and incomplete questionnaires with missing responses were excluded from the study.

### Measures

The online survey was a self-administered questionnaire that took 10 to 12 minutes to complete. It consisted of two sections: (a) basic sociodemographic characteristics and (b) measurements.

The first section collected crucial data on the participants' sociodemographic and work-related backgrounds. This encompassed factors such as gender (male, female, or prefer not to mention), age, scientific degree (Doctor of Science, PhD, or Magister degree), and professional role (Professor, Associate Professor, Senior Lecturer, or Assistant). Additionally, war-related variables included migration processes (external military migrants, internal military migrants, or permanent location) and information about university placement (permanent location or temporarily relocated to Ukraine-controlled territory).

The second section comprised a self-reported Maslach Burnout Inventory-Human Services Study (MBI-HSS), which was adapted specifically for socioeconomic professions by Vodopyanova. The MBI-HSS questionnaire was chosen due to its well-established validity and reliability, comprehensive assessment of burnout, and widespread acceptance in the field, making it a preferred tool for studying burnout. Furthermore, the MBI-HSS has been translated and adapted for Ukrainian cultural contexts, making it suitable for this study. This inventory comprises 22 items and measures three predictors of burnout: emotional exhaustion (EE), depersonalization (DP), and personal accomplishment (PA)^17^. The MBI-HSS measures the frequency of manifestations of each aspect of burnout, and response options range from 'every day' to 'never'. The responses are grouped by category and summed to assess each category, following a standardized procedure for counting and analyzing the respondents' responses.

### Statistical analysis

The data generated through Google Forms was downloaded into an Excel spreadsheet and imported into the SPSS^®^ software (Statistical Package for the Social Sciences) for analysis. There were no missing data as answering all questions was mandatory.

To test the hypothesis of normal distribution, we used the Kolmogorov-Smirnov test with Lilliefors significance correction for large sample sizes and the Shapiro-Wilk test for small sample sizes (< 50).

The analysis of the data obtained from the first and second cuts (Table 4) led us to conclude that not all the data are normally distributed, as the condition p < 0.05 is not met in some cases.

**Table 4:** Tests of normality.

Burnout Predictor	Man						Woman					
	Kolmogorov-Smirnov^a^, 1st wave			Shapiro-Wilk, 2nd wave			Kolmogorov-Smirnov^a^, 1st wave			KolmogorovSmirnov^a^, 2nd wave		
	Statistic	df	Sig.	Statistic	df	Sig.	Statistic	df	Sig.	Statistic	df	Sig.
EE	0.057	171	0.200*	0.954	31	0.199	0.044	665	0.004	0.041	197	0.200*
DP	0.128	171	0.000	0.970	31	0.528	0.106	665	0.000	0.104	197	0.000
PA	0.062	171	0.200*	0.968	31	0.467	0.052	665	0.000	0.076	197	0.008

*EE* emotional exhaustion, *DP* depersonalization, *PA* personal accomplishment.

^a^Lilliefors Significance Correction.

*This is a lower bound of the true significance.

Some of the data had a distribution that differed from the normal distribution, specifically 1_m_, 1_m_, 2_m_, 2_m_DP, 2_m_PA, 2_w_EE. These conclusions were confirmed by analyzing graphs and values for skewness and kurtosis. Therefore, for statistical calculations, we utilized the nonparametric Kruskal Wallis test, which does not necessitate a normal distribution of the underlying data.

We conducted separate analyses to determine whether age, possession of a scientific degree, position in the workplace, relocation of the university to a new location, and change of location since the full-scale war in Ukraine had an influence on DP and PA of men and women during the 1st and 2nd cuts.

The assumptions required for performing the Kruskal-Wallis test were checked:

The independent variables consisted of two or more categorical groups, including age (divided into four groups: up to 35 years, 35–45 years, 46–60 years, and 60 years and above), scientific degree (divided into three groups: doctor of sciences, PhD, and without a scientific degree), position at the workplace (divided into four groups: professor, associate professor, senior lecturer, and assistant), current university location (divided into two groups: yes, the university was relocated; no, the university was not relocated), and change of permanent residence (internal military migrants, external military migrants, remained in the places of permanent residence).

The dependent variables were measured at low, moderate, and high levels of EE, DP, and RA for both men and women.

There was no relationship between observations within each group or between the groups themselves, indicating that the samples were independent.

The score distribution for each group of the independent variable did not have the same shape, thus requiring a comparison of average ranks.

The hypothesis H0 was accepted, indicating that the independent variables do not have an influence on DP and PA of both men and women. On the other hand, the hypothesis H1 was also considered, indicating that the independent variables have an influence on EE and DP of both men and women. A statistical significance level of p<0.05 was used.

### Ethical considerations

In conducting the research, all methods and procedures were performed in strict accordance with the relevant guidelines and regulations. This includes adherence to the standards of the Berdyansk State Pedagogical University, the legal requirements of Ukraine, and the ethical guidelines for research involving human subjects, as specified in the Declaration of Helsinki. Furthermore, the research was designed and conducted in accordance with the guidelines provided by the Scientific Reports journal. This has been confirmed by the Ethics Committee of Berdyansk State Pedagogical University in their approval of the study under protocol number 05/2022.

### Ethical considerations

The Ethics Committee of Berdyansk State Pedagogical University approved the study conducted under protocol number 05/2022, ensuring its safety and fairness. Participants were fully informed of their voluntary participation and their right to withdraw at any time without providing an explanation. The study design prioritized the protection of the participants and did not pose any risks to them. All the information collected was kept anonymous and confidential. Informed consent was obtained digitally through Google Forms. No incentives were offered to participants.

## Data Availability

The data sets used and/or analyzed during the current study are available from the corresponding author on reasonable request.
